# Combined use of lysyl oxidase, carcino-embryonic antigen, and carbohydrate antigens improves the sensitivity of biomarkers in predicting lymph node metastasis and peritoneal metastasis in gastric cancer

**DOI:** 10.1007/s13277-014-2355-5

**Published:** 2014-07-25

**Authors:** Hao Lai, Qinwen Jin, Yuan Lin, Xianwei Mo, Bo Li, Ke He, Jiansi Chen

**Affiliations:** 1 Graduate College, Guangxi Medical University, Nanning, 530021 Guangxi Autonomous Region China; 2 Department of Gastrointestinal Surgery, Affiliated Cancer Hospital of Guangxi Medical University, 71 Hedi Road, Nanning, 530021 Guangxi Autonomous Region China; 3 Department of Head and Neck Surgery, Affiliated Cancer Hospital of Guangxi Medical University, Nanning, 530021 Guangxi Autonomous Region China

**Keywords:** Gastric cancer, Lysyl oxidase, Predictive value, Biomarker

## Abstract

The purpose of this study was to determine whether lysyl oxidase (LOX) is a useful marker of metastasis in gastric cancer (GC) patients in combination with tumor markers carcino-embryonic antigen (CEA), carbohydrate antigen 724 (CA724), carbohydrate antigen 19-9 (CA19-9), and carbohydrate antigen 125 (CA125). There were 215 GC patients (67 without metastasis, 102 with lymph node metastasis, and 46 with peritoneal metastasis) who presented to the Affiliated Cancer Hospital of Guangxi Medical University between May 2009 and November 2012 that were enrolled in this study. The LOX expression level and the serum concentration of the four tumor markers were evaluated preoperatively. All patients underwent computed tomography (CT) and ultrasonography (US) before surgery. Statistical analysis, including receiver operating characteristic (ROC) curve analysis, area under the curve (AUC) analysis, and logistic regression analysis, was performed to evaluate the diagnostic value of these markers in predicting metastasis in GC. For predicting lymph node metastasis in GC, the sensitivity of LOX, CEA, CA724, CA199, and CA125 was 44.12, 12.75, 21.57, 23.53, and 15.69 %, respectively, and increased to 79.41 % in combination. For predicting peritoneal metastasis in GC, the sensitivity of these markers was 56.52, 23.91, 34.78, 36.96, and 34.78 %, respectively, and increased to 91.30 % in combination. Combining LOX with CEA, CA724, CA199, and CA125 could increase the sensitivity of predicting lymph nodes metastasis and peritoneal metastasis in GC. Surgeons can use these markers to determine the best treatment options for patients. Additional large-scale, prospective, multicenter studies are urgently needed to further confirm the results of this study.

## Introduction

Gastric cancer (GC) is the fourth most common cancer and the second leading cause of cancer deaths worldwide [1]. Nearly half of GC cases occur in China, with an overall 5-year survival rate of approximately 20 % [2]. Most GC cases are diagnosed in advanced stages [3], and thus the opportunity for radical surgery is lost. Lack of early detection and limited treatment options contribute to the poor prognosis in GC [4]. As the prognosis of GC patients is closely related to timely diagnosis and appropriate treatment, an effective tumor biomarker is urgently needed for screening and diagnosis [5]. Advances in basic research and molecular biology mean that it should now be possible to detect effective tumor biomarkers to diagnose GC [6], thereby improving treatment options for patients with advanced GC metastasis.

Lysyl oxidase (LOX) is a copper-dependent amine oxidase encoded by members of a five-gene family that includes LOX and four LOX-like proteins (LOXL 1–4) [7]. LOX controls both the structure and the tensile strength of the extracellular matrix and thus preserves tissue integrity [8]. Numerous studies have highlighted the role of LOX as a marker of tumor progression and metastasis, such as in bronchogenic carcinoma and in breast cancer, colorectal cancer, and ovarian cancer [9–12]. However, to the best of our knowledge, no studies have investigated the correlation of LOX expression and it predicts information for metastasis in GC patients, in condition of combine LOX with other tumor markers, such as carcino-embryonic antigen (CEA), carbohydrate antigen 724 (CA724), carbohydrate antigen 19-9 (CA19-9), and carbohydrate antigen 125 (CA125).

The present study analyzed the association between LOX expression and its diagnostic significance for metastasis GC, in condition of combine LOX with serum tumor markers CEA, CA724, CA125, and CA199.

## Materials and methods

### Patients and tissue samples

This study was approved by the Research Ethics Committee of the Affiliated Cancer Hospital of Guangxi Medical University in China. There were 215 patients with GC who were diagnosed in the hospital between May 2009 and November 2012 that were enrolled in this study. None of the patients had received preoperative adjuvant chemotherapy or radiotherapy. Written informed consent was obtained from all the patients. Fresh GC specimens were obtained by preoperative gastroscopy and were fixed in 10 % formalin and embedded in paraffin, and pathological examination was performed. Further postoperative pathological analysis was done for surgery patients. All the specimens were handled and anonymized according to ethical and legal standards. All the GC patients underwent diagnostic imaging with computed tomography (CT) or ultrasonography (US) prior to the surgery.

According to the pathology report, the GC patients were divided into the following groups based on their degree of metastasis: (1, GC patients without metastasis; 2, advanced GC with lymph node metastasis; and 3, advanced GC with peritoneal metastasis.

### Immunohistochemistry

**Fig. 1:**
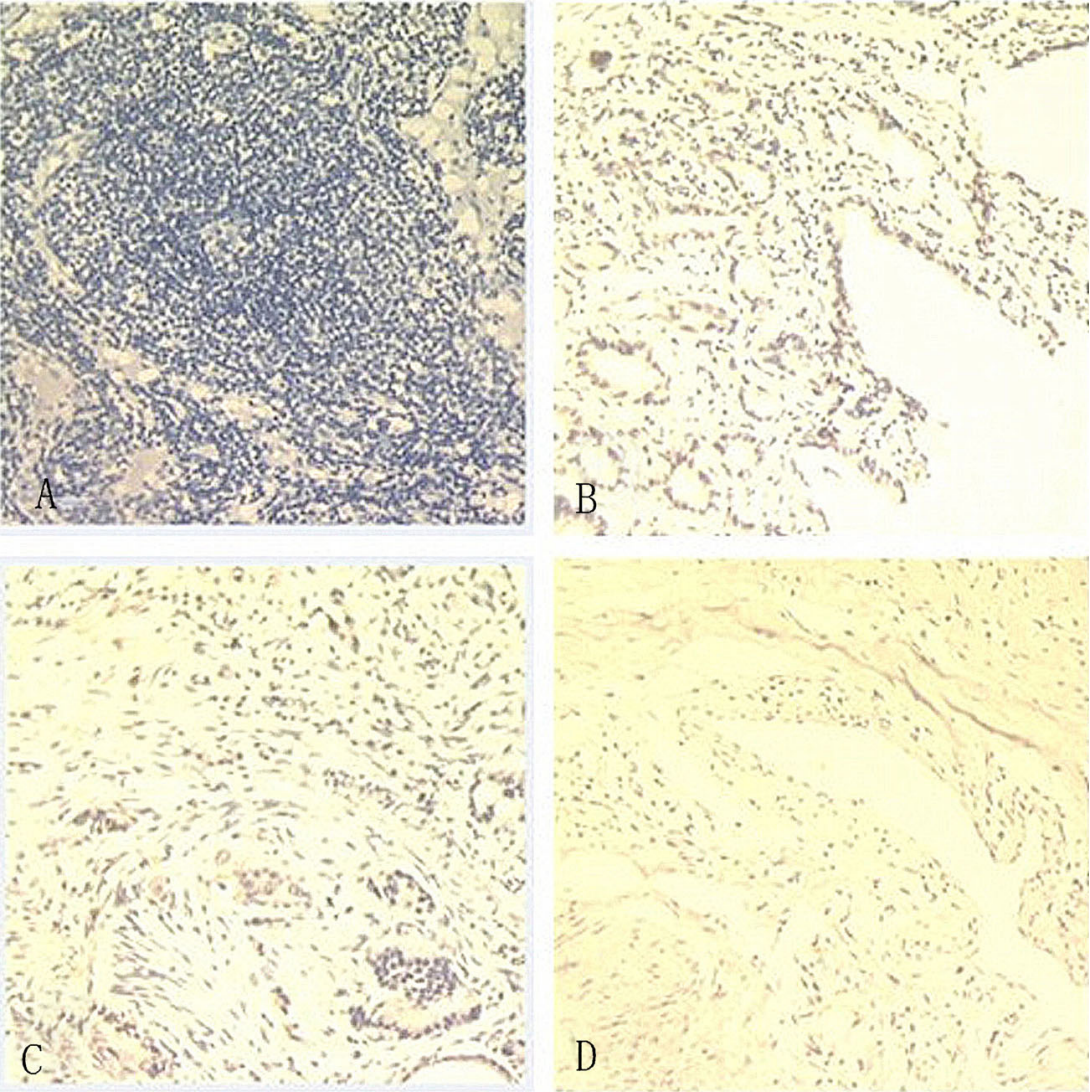
**a** Negative expression of lysyl oxidase (LOX), **b** weak expression of LOX, **c** moderate expression of LOX, and **d** strong expression of LOX

The expression pattern of LOX in tissue samples was analyzed with the labeled streptavidin-peroxidase immunohistochemical (IHC) technique. Tissue slides were deparaffinized in xylene and rehydrated in graded series of ethanol, followed by heat-induced epitope retrieval in citrate buffer (pH 6.0). LOX expression was detected using a primary antibody against LOX (anti-LOX antibody, rabbit polyclonal to LOX, 1/300; Abcam, Cambridge, MA, USA). The degree of immunostaining was reviewed and scored by two pathologists, taking into account the percentage of positive cells and the staining intensity, as described by Hu et al. [13]. The immunostaining was classified into four groups, with the proportion of cell protein expression categorized as follows [13]: 0–10 % was recorded as 0, 10–30 % was recorded as 1, 30–50 % was recorded as 2, 50–75 % was recorded as 3, and >75 % was recorded as 4. Cell protein expression was then graded according to the sum of the scores: 1, Fig. 1a, negative expression (−, score of 0–1); 2, Fig. 1b, weak expression (+, score of 2–3); 3, Fig. 1c, moderated expression (++, score of 4–5); and 4, Fig. 1d, strong expression (+++, score of 6–7).

Blood samples were collected from each patient within 5–7 days before the surgery, and CEA levels were tested with a fluorescence-enzyme immunoassay. CA724, CA125 (Fujirebio Diagnostics, PA, USA), and CA19-9 (Immunotech, Marseille, France) were also measured with an immunoradiometric assay. The cut-off values for CEA, CA72-4, CA19-9, and CA125, were defined as 5.0 ng/ml, 5 U/ml, 37 U/ml, and 35 U/ml, respectively, according to literature reports on a Chinese population and the manufacturer’s instructions [14–16].

### Statistics

The Chi-square test was used to evaluate the association between LOX expression and age, gender, tumor location, differentiation, depth of invasion, metastasis status. The area under the curve (AUC) of the receiver operating characteristic (ROC) curve was used to evaluate the predictive value of LOX, CEA, CA724, CA199, and CA125 for GC with different metastasis status. Multivariate logistic regression analysis was used to establish the diagnostic mathematical model. On the basis of this model, the prediction value was calculated, followed by ROC curve analysis. The statistical analysis was performed with the Statistical Package for the Social Sciences, version 16.0 (SPSS 16.0), with a *P* < 0.05 considered to be significant.

## Results

The IHC results revealed that 90 of the 215 (41.86 %) GC patients had different expression levels of LOX. The LOX expression pattern and clinic pathological factors are listed in Table 1. The LOX expression pattern was significantly correlated with tumor metastasis status (*P* < 0.05), but it was not associated with age, gender, tumor location, differentiation, and depth of invasion (*P* > 0.05). Overall, the sensitivity of LOX for predicting metastasis in GC (lymph node metastasis and peritoneal metastasis) was 47.97 %. For predicting lymph node metastasis in GC, the sensitivity of LOX expression was 44.12 %, and this increased to 56.52 % for predicting peritoneal metastasis.

**Table 1:** Relationship between LOX expression and clinicopathological factors in GC patients

Characteristics	Sample size (*n*)	LOX (−) *n* (%)	LOX (+) *n* (%)	LOX (++) *n* (%)	LOX (+++) *n* (%)	*P* value
*Age*						
<55 year	109	68	9	17	15	0.21
≥55 year	106	51	12	24	19	
*Gender*						
Male	95	49	13	16	17	0.26
Female	120	70	8	25	17	
*Tumor location*						
Pylorus	70	37	6	13	14	0.33
Gastric corpus	54	26	8	9	11	
Gastric fundus	91	56	7	19	9	
*Differentiation*						
Poor	96	58	9	16	13	0.61
Well + intermediate	109	61	12	25	11	
*Depth of invasion*						
Mucosa	28	16	5	5	2	0.35
Muscular layer	42	21	6	7	5	
Serosa	145	82	10	29	27	
*Metastasis status*						
Without metastasis	67	48	8	7	4	0.00
LN metastasis	102	57	9	25	11	
Distance metastasis	46	20	4	7	15	

In all the GC patients, preoperative levels of CEA, CA724, CA19-9, and CA125 were above the cut-off levels (13.49, 21.40, 23.72, and 18.60 %, respectively). The effect estimates of diagnostic tests of the different markers are shown in Table 2 and Table 3. In predicting lymph node metastasis in GC, CA199 had the highest sensitivity (23.53 %), specificity (85.07 %), and accuracy (47.93 %) among the four serum tumor markers, and CEA had the worst sensitivity (12.75 %), specificity (92.54 %), and accuracy (44.38 %). In GC patients with peritoneal metastasis, CA199 had the highest sensitivity (36.96 %), and CEA had the lowest sensitivity (23.91 %). As the degree of metastasis increased, the positive rate of serum CEA, CA724, CA199, and CA125 increased. The sensitivity of the diagnostic imaging (CT or US) in lymph node metastasis patients and peritoneal metastasis patients was low, (7.84 and 15.22 %, respectively). The positive likelihood ratio and negative likelihood ratio of these markers in detecting different metastasis in GC are also presented in Table 2 and Table 3.

**Table 2:** Predictive ability of different markers for lymph node metastasis gastric cancer

Tumor marker	AUC (95 % CI)	Sensitive	Specificity	Accuracy	PLR	NLR
LOX	0.599^a^ (0.514–0.685)	44.12 %	71.64 %	55.03 %	1	0.84
CEA	0.562^a^ (0.475–0.649)	12.75 %	92.54 %	44.38 %	0.81	0.92
CA724	0.569^a^ (0.483–0.655)	21.57 %	88.06 %	47.92 %	0.92	0.90
CA199	0.663^a^ (0.578–0.748)	23.53 %	85.07 %	47.93 %	0.89	0.91
CA125	0.574^a^ (0.486–0.661)	15.69 %	88.06 %	44.38 %	0.67	0.97
Combination	0.682 (0.602–0.763)	79.41 %	31.34 %	76.33 %	2.79	0.55

*AUC* area under the curve, *CI* confidence interval, *PLR* positive likelihood ratio, *NLR* negative likelihood ratio

^a^ *P* < 0.05 compared with combination of all markers

**Table 3:** Predictive ability of different marker for peritoneal metastasis gastric cancer

Tumor marker	AUC (95 % CI)	Sensitive	Specificity	Accuracy	PLR	NLR
LOX	0.639^a^ (0.542–0.736)	56.52 %	62.13 %	60.93 %	0.41	0.19
CEA	0.741^a^ (0.667–0.815)	23.91 %	89.35 %	75.35 %	0.62	0.23
CA724	0.689^a^ (0.607–0.772)	34.78 %	82.25 %	72.09 %	0.53	0.22
CA199	0.690^a^ (0.605–0.775)	36.96 %	79.88 %	70.70 %	0.50	0.21
CA125	0.754^a^ (0.671–0.836)	34.78 %	85.80 %	74.88 %	0.67	0.21
Combination	0.787 (0.717–0.858)	91.30 %	20.59 %	29.30 %	0.52	0.14

*AUC* area under the curve, *CI* confidence interval, *PLR* positive likelihood ratio, *NLR* negative likelihood ratio

^a^ *P* < 0.05 compared with combination of all markers

**Fig. 2:**
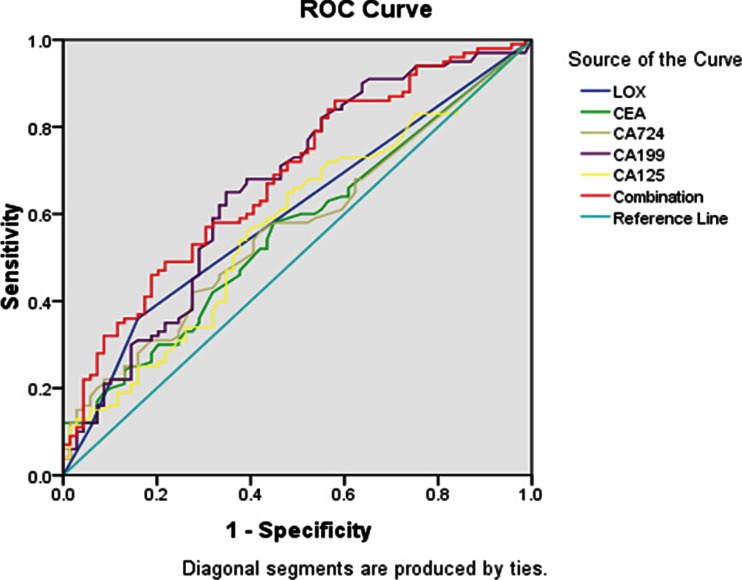
Receiver operating characteristic (*ROC*) curve of single and combined markers in predicting lymph node metastasis in gastric cancer (GC)

**Fig. 3:**
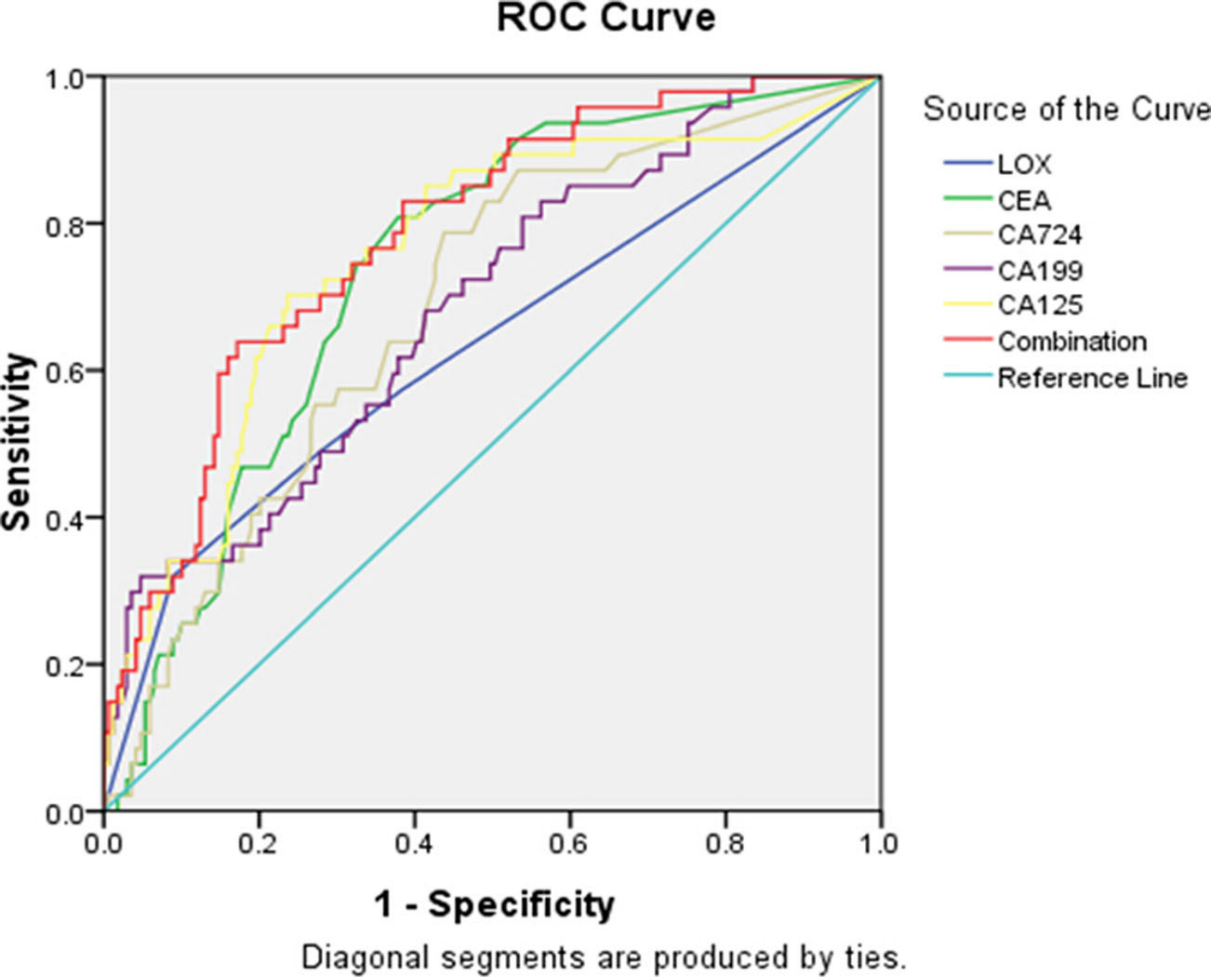
Receiver operating characteristic (*ROC*) curve of single and combined markers in predicting peritoneal metastasis in gastric cancer (GC)

As the sensitivity of a single serum tumor marker in predicting metastasis in GC was low, its potential in clinical application would be limited. Therefore, we analyzed the sensitivity when these markers were combined and obtained the AUC of ROC curve. We then calculated their diagnostic values in GC with different metastasis status. The combined markers yielded an ROC value of 0.682, which was significantly higher than that of the single marker (*P* < 0.05) and better able to distinguish lymph node metastasis in GC (Table 2 and Fig. 2). In peritoneal metastasis patients, the ROC value of the five markers combined was 0.787, higher than each single marker (Table 3 and Fig. 3).

## Discussion

Metastasis is one of the main causes of death in patients with GC tumors [17]. Early detection of metastasis and appropriate treatment are of critical importance for patient outcomes. For example, surgical resection with extensive lymphadenectomy was shown to result in better outcomes in GC involving the lymph nodes [18], and the positive effect of neoadjuvant intraperitoneal and systemic chemotherapy on patients with advanced GC and peritoneal dissemination has been demonstrated [19, 20].

CT and US can help to predict metastasis GC, but many studies have shown that these are not reliable indicators of metastasis [21, 22]. Our data showed that the sensitivity of these diagnostic imaging modalities in lymph node metastasis patients and peritoneal metastasis patients was only 7.84 and 15.22 %, respectively. The predictive value of PET/CT was high in local lymph node metastasis and distant metastasis in GC patients [23]. However, it is costly, and most patients are unable to afford the procedure. Laparoscopic exploration is less invasive than open surgery for diagnosing malignant abdominal disease [24]. However, it is also costly and time-consuming, and surgeons are reluctant to undertake it. Several of the most frequently used tumor markers, such as CEA, CA724, CA199, and CA125, provide additional diagnostic information in gastrointestinal malignancies [25, 26], but the sensitivity of any one marker alone is not sufficient [27]. In our group, the sensitivity of CEA, CA724, CA199, and CA125 in the GC patients with lymph node metastasis was only 12.75, 21.57, 23.53, and 15.69 %, respectively. In the peritoneal metastasis patients, the sensitivity of these four markers was 23.91, 34.78, 36.96, and 34.78 %, respectively. As the diagnosis of GC is most often performed when the tumor is at an advanced stage [28], there is an urgent need to identify new markers (diagnostic methods) to provide appropriate treatment and improve prognoses.

LOX was initially reported as a copper-dependent amine oxidase responsible for the catalysis of collagen and elastin cross-linking within the extracellular matrix [29]. A recent study highlighted the role of LOX family oxidases in promoting cancer metastasis [30]. LOX is highly expressed in invasive tumors, such as uveal melanoma, colorectal cancer, and gastric cancer, and it is closely associated with metastasis and poor patient outcomes [12, 29, 31]. Our study demonstrates that increased expression of LOX is correlated with an advanced stage of GC and that it may contribute to tumor development. This finding is consistent with that of Zhang et al. [29]. In lymph node metastasis and peritoneal metastasis in GC, the rate of LOX overexpression was 44.12 and 56.52 %, respectively, in the current study. Therefore, LOX is a correlative biomarker of metastasis in GC.

However, based our results, the sensitivity and accuracy of LOX alone are limited (around 50 % and no more than 61 %, respectively). Therefore, the use of LOX alone does not meet the requirements of clinical practice. Several studies found that a combination of different tumor marker may improve diagnostic accuracy in gastrointestinal tract malignancies compared with single biomarkers alone. For example, Emoto et al. [32] showed that the combined use of CEA, CA199, CA725, and CA125 may improve the sensitivity of these biomarkers in detecting peritoneal metastasis in GC. Chen et al. [33] revealed that combining CA724 with CEA and CA199 considerably improves the sensitivity of these biomarkers in detecting GC, without impairing specificity. The choice of serum tumor markers to be combined with LOX requires further investigation to determine how to improve the sensitivity of these biomarkers in the detection of metastasis in GC.

We carefully selected other serum tumor markers correlated with tumor invasion and combined these with LOX to improve the sensitivity of these in detecting metastasis in GC. Several studies revealed that CA724 and CA199 are correlated with invasive GC, lymph node involvement, and tumor stage [34–40] and that combined use of CEA with CA724 and CA199 considerably improves the positive rate, without impairing the specificity [41]. However, our results showed that the preoperative positivity of CEA, CA724, CA19-9, and CA125 was extremely low, making it a poor biomarker of lymph node metastasis and peritoneal metastasis in GC. When we combined all the markers, their sensitivity in detecting lymph node metastasis in GC was 79.41 %. The sensitivity for GC with peritoneal metastasis was 91.30 %, which was higher than when a single marker was used (Table 2 and Table 3). The ROC curve analysis also revealed that the combination of all markers yielded a value of 0.682 for GC with lymph node metastasis and 0.787 for GC with peritoneal metastasis. These values were significantly higher than the sensitivity with one marker (*P* < 0.05, Table 2 and Table 3).

Interestingly, our study showed that in the GC patients with lymph node metastasis, CA125 was positive in only 15.69 % of cases, but it was positive in 34.78 % of GC cases with peritoneal metastasis. This finding is consistent with that reported in a study by Emoto et al. [32], who found that CA125 was correlated with the degree of peritoneal dissemination in GC and that it was highly sensitive in predicting peritoneal metastasis.

We did not evaluate other tumor markers, such as carbohydrate antigen 50, alpha fetal protein (AFP), and carbohydrate antigen 242, because these markers are not commonly measured in GC patients, and very few studies have shown any association between these markers and lymph node or peritoneal metastasis in GC. For example, most AFP-positive GC was correlated with liver metastasis [33].

In summary, we found that LOX is a correlative tumor biomarker for GC with lymph node metastasis and peritoneal metastasis in a Chinese population. The combined use of LOX with other markers (LOX + CEA + CA724 + CA199 + CA125) could improve their sensitivity in predicting metastasis in GC.

Our study has several limitations. First, this is a retrospective analysis with a relatively small sample from a single institute. A large, prospective, multicenter study is needed to demonstrate the predictive value of LOX in GC metastasis in combination with other tumor markers. Second, we could not accurately distinguish the metastasis N stage and the peritoneal dimensional status (*P*_0_, *P*_1_, *P*_2_, and *P*_3_) because LOX expression was evaluated by qualitative detection, not by quantitative determination, and our sample size was not large. Third, uncontrolled or unmeasured confounding factors, such as selection bias in GC patients and potential laboratory errors in evaluating LOX expression, may have produced bias.

## Conclusions

The combined use of LOX with CEA, CA724, CA199, and CA125 could increase the sensitivity of predicting lymph nodes metastasis and peritoneal metastasis in GC. Surgeons can use these markers to determine the best treatment options for patients. Additional large-scale, prospective, multicenter studies are urgently needed to further confirm the results of this study.
